# Hijacking 5-Fluorouracil Chemoresistance in Triple Negative Breast Cancer via microRNAs-Loaded Chitosan Nanoparticles

**DOI:** 10.3390/ijms25042070

**Published:** 2024-02-08

**Authors:** Sherif Ashraf Fahmy, Noha Khalil Mahdy, Adham H. Mohamed, Fatma A. Mokhtar, Rana A. Youness

**Affiliations:** 1 Chemistry Department, School of Life and Medical Sciences, University of Hertfordshire Hosted by Global Academic Foundation, New Administrative Capital, Cairo 11835, Egypt; sheriffahmy@aucegypt.edu; 2 Department of Pharmaceutics and Industrial Pharmacy, Faculty of Pharmacy, Cairo University, Kasr El-Aini Street, Cairo 11562, Egypt; nkmahdy@gmail.com; 3 Biology and Biochemistry Department, Molecular Genetics Research Team (MGRT), Faculty of Biotechnology, German International University (GIU), New Administrative Capital, Cairo 11835, Egypt; 4 Department of Chemistry, Faculty of Science, Cairo University, Giza 12613, Egypt; 5 Fujairah Research Centre, Sakamkam Road, Fujairah 1626, United Arab Emirates; 6 Department of Pharmacognosy, Faculty of Pharmacy, El Saleheya El Gadida University, El Saleheya El Gadida 44813, Sharkia, Egypt

**Keywords:** miR-1275, miR-615-5p, Let-7i, 5-Fluorouracil, triple-negative breast cancer, chemoresistance, chitosan nanoparticles

## Abstract

Chemotherapy is still the mainstay of treatment for triple-negative breast cancer (TNBC) patients. Yet only 20% of TNBC patients show a pathologic complete response (pCR) after neoadjuvant chemotherapy. 5-Fluorouracil (5-FU) is a stable cornerstone in all recommended chemotherapeutic protocols for TNBC patients. However, TNBC patients’ innate or acquired chemoresistance rate for 5-FU is steeply escalating. This study aims to unravel the mechanism behind the chemoresistance of 5-FU in the aggressive TNBC cell line, MDA-MB-231 cells, to explore further the role of the tumor suppressor microRNAs (miRNAs), miR-1275, miR-615-5p, and Let-7i, in relieving the 5-FU chemoresistance in TNBC, and to finally provide a translational therapeutic approach to co-deliver 5-FU and the respective miRNA oligonucleotides using chitosan-based nanoparticles (CsNPs). In this regard, cellular viability and proliferation were investigated using MTT and BrdU assays, respectively. 5-FU was found to induce JAK/STAT and PI3K/Akt/mTOR pathways in MDA-MB-231 cells with contaminant repression of their upstream regulators miR-1275, miR-615-5p, and Let-7i. Moreover, CsNPs prepared using the ionic gelation method were chosen and studied as nanovectors of 5-FU and a combination of miRNA oligonucleotides targeting TNBC. The average particle sizes, surface charges, and morphologies of the different CsNPs were characterized using dynamic light scattering (DLS) and transmission electron microscopy (TEM), respectively. In addition, the encapsulation efficiency (EE%), drug loading capacity (DLC%), and release manner at two different pH values were assessed. In conclusion, the novel CsNPs co-loaded with 5-FU and the combination of the three miRNA oligonucleotides demonstrated synergistic activity and remarkable repression in cellular viability and proliferation of TNBC cells through alleviating the chemoresistance to 5-FU.

## 1. Introduction

Breast cancer (BC) is the most prevalent malignancy and the primary reason for cancer-related mortalities among females [1]. In 2020, there were 2.3 million new BC cases, making up 11.7% of all new cancer cases; 684,996 cases ended in death [2,3]. BC has been categorized as one of the most heterogeneous cancers, with at least 18 distinct subtypes of BC identified by the World Health Organization (WHO) [1,4,5]. One of the most violent BC subtypes is triple-negative breast cancer (TNBC) [6]. TNBC is distinguished by the absence of estrogen (ER) and progesterone (PR) receptor expression, as well as the absence of human epidermal growth factor receptor 2 (HER-2) overexpression. Unfortunately, TNBC is more prevalent among younger females, particularly those of African ancestry [7].

Chemotherapy is the mainstay of treatment for TNBC patients [8]. Yet, some TNBC patients (almost 20%) show a pathologic complete response (pCR) after neoadjuvant chemotherapy [9]. According to National Comprehensive Cancer Network (NCCN) recommendations, the following systemic chemotherapy regimens are available for TNBC patients: Docetaxel and Cyclophosphamide (TC), Taxel/Docetaxel, Adriamycin, and Cyclophosphamide (TAC), Adriamycin and Cyclophosphamide (AC), Cyclophosphamide, Methotrexate, and 5-Fluorouracil (CMF), Cyclophosphamide, Adriamycin, and 5-Fluorouracil (CAF), and Cyclophosphamide, Epirubicin, 5-Fluorouracil, and Paclitaxel/Docetaxel (CEF-T) [8,10], highlighting the fundamental role of 5-Fluorouracil (5-FU) as a stable cornerstone in all recommended chemotherapeutic protocols available for TNBC patients.

As previously mentioned, 5-FU is a widely used chemotherapeutic drug in several solid malignancies, including TNBC [11]. In 5-FU, the hydrogen atom at the C-5 position has been replaced with a fluorine atom to make it an analog of uracil. The same assisted transport system that uracil uses helps it enter the cell quickly. 5-FU is converted into many active metabolites, which are fluorodeoxyuridine monophosphate (FdUMP), fluorodeoxyuridine triphosphate (FdUTP), and fluorouridine triphosphate (FUTP). These metabolites inhibit thymidylate synthase and disrupt DNA and RNA synthesis through misincorporation mechanisms [12]. On the other hand, 5-FU showed a poor pharmacokinetic profile, as evidenced by its low bioavailability, short half-life time, and drug resistance [13].

TNBC patients are the least fortunate not only because of their poor prognosis, late diagnosis, and aggressive nature of the tumors but also because of their high rates of chemoresistance [14,15]. TNBC patients might acquire resistance after various cycles of chemotherapy or have a natural tendency to be less susceptible to chemotherapeutic protocols [16]. On the molecular level, the chemoresistance might be attributed to several reasons, such as hypoxia and overexpression of ATP-binding cassette (ABC) transporters by cancer stem cells. Yet, the most predominant resistance mechanism in African TNBC patients is the overexpression and/or hyperactivation of several oncogenic signaling cascades, such as the PI3K/AKT/mTOR and JAK/STAT pathways [17,18,19].

PI3K/AKT/mTOR pathway hyperactivation is common in TNBC, owing primarily to PTEN (tumor suppressor phosphatase and tensin homolog) loss, a negative regulator for the PI3K/AKT/mTOR pathway, which is directly linked with an unfavorable clinical course of the disease, aggressive TNBC tumors, and poor survival of patients [5,20]. Yet, mTOR inhibition reduced the resistance in several cancer cells, including BC cells, and increased their susceptibility to chemotherapeutic drugs [17,21,22,23].

Similarly, the JAK/STAT pathway is noticeably expressed in TNBC and is associated with aggressive clinical behavior, adverse outcomes, and chemotherapeutic resistance [17,24,25]. STAT3 is a playmaker in such association with chemoresistance as it interacts with NF-kB, resulting in chemoresistance. Furthermore, STAT3 induces HIF1α expression, thus proving its involvement in hypoxia-mediated chemoresistance in TNBC [17,26].

MicroRNAs (miRNAs) have been cast as potential regulators of the PI3K/AKT/mTOR and JAK/STAT pathways in several malignant contexts [1,18,23,27,28,29]. In this regard, miRNAs have been extensively reported to play a multi-player role in simultaneously hijacking several oncogenic singling cascades and thus hold tremendous therapeutic potential for several malignancies [15,30,31]. Nonetheless, miRNAs were reported to play a significant role in TNBC chemoresistance and re-sensitizing TNBC tumors to conventional chemotherapeutic agents. Some of them were through regulating the PI3K/AKT/mTOR and JAK/STAT pathways [32,33,34,35]. Among the most prominent miRNAs that were validated to either directly or indirectly affect PI3K/AKT/mTOR and JAK/STAT pathways are the tumor suppressor miRNAs: miR-1275 [36], Let-7i [37], miR-147 [38], miR-486-5p [11,27,28], miR-615-5p [39,40], and the oncomiR-155 [41,42]. This array of miRNAs showed great potential for tuning BC oncogenic and immunogenic profiles.

Yet, the clinical translation of miRNAs has been proven to be quite challenging due to their untargeted delivery, off-target effects, immunogenicity, quick degradation in the blood circulation by nucleases, and short half-life in systemic circulation [1,23,43,44,45]. The evolution of nanomedicine has positively rebranded RNA-based therapeutics, where most RNA-mediated challenges are solved using a suitable-architectured nano-based delivery system [46,47,48,49,50,51].

One of the widely used nanomaterials is chitosan nanoparticles (CsNPs), a widely accepted hydrophilic natural polymer characterized by its biocompatibility, biodegradability, and mucoadhesion properties [51]. CsNPs are positively charged carriers that can be used as nanovectors to carry the 5-FU and miRNA oligonucleotides via electrostatic interaction, enhancing their cellular uptake [52,53]. In addition, the hemocompatibility and high stability of CsNPs in different biological fluids, such as human plasma and serum, and hence their enhanced therapeutic activity, have been reported. This high stability in biological fluids is attributed to the deswelling of the CsNPs at physiological pH, driven by the decline of the intramolecular electric repulsions inside the polymeric membranes [54,55].

Therefore, this study aims to unravel the mechanism of 5-FU resistance in MDA-MB-231 TNBC cells, provide a molecular approach to alleviate 5-FU chemoresistance using miRNAs of choice, and finally co-deliver 5-FU and respective miRNA oligonucleotides using CsNPs in a translational approach to overcome the poor bioavailability and low cellular uptake profiles of loaded cargos.

## 2. Results and Discussion

### 2.1. MDA-MB-231 TNBC Cell Shows Resistance to 5-FU

**Figure 1:**
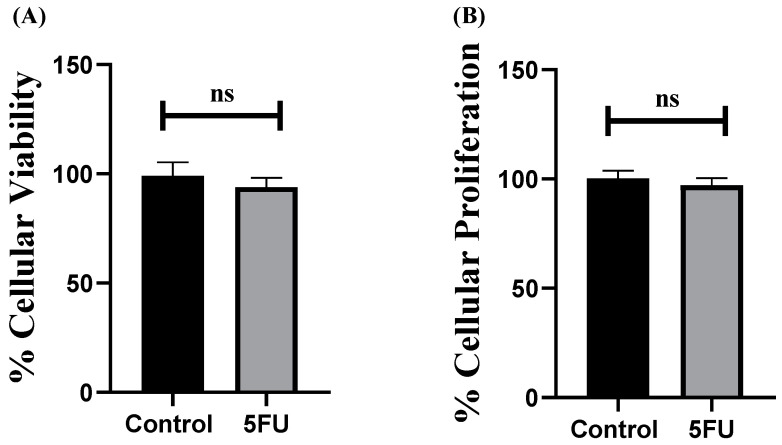
Impact of 5-FU on cellular viability and proliferation in MDA-MB-231 cells. (**A**) Cellular viability was assessed using MTT assay; 5-FU resulted in a non-significant alteration in cellular viability compared with control cells. (**B**) Cellular proliferation rate was assessed using the BrdU incorporation assay. 5-FU resulted in a non-significant alteration in cellular proliferation of MDA-MB-231 cells compared with control cells. Data are presented as the mean ± SEM of three independent experiments. ^ns^ *p* > 0.05.

First, the sensitivity of MDA-MB-231 cells to 5-FU was evaluated by measuring cellular viability and proliferation upon treating the cells with 5-FU for 72 h. The results showed that 5-FU had non-significant effects on MDA-MB-231 cellular viability and proliferation compared to control cells (Figure 1), proving the resistance of MDA-MB-231 cells to 5-FU. This goes in line with other studies showing that 5-FU showed similar resistance in HER 2^+^ cells, MDA-MB-543, and SKBR-3 cell lines [56]. Nonetheless, several reports recently reported the innate [17] and/or acquired [57] resistance of TNBC patients to 5-FU.

### 2.2. 5-FU Induces the Activation of JAK/STAT and PI3K/Akt/mTOR Pathways in MDA-MB-231 Cells

**Figure 2:**
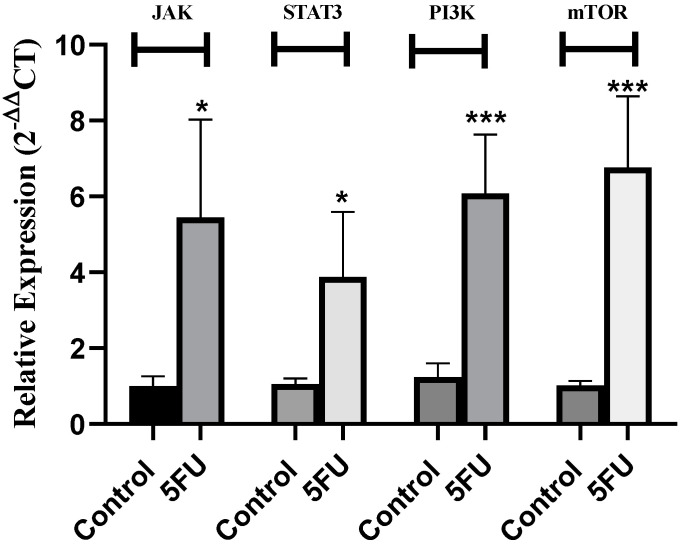
Impact of 5-FU on JAK/STAT and PI3K/Akt/mTOR pathways in MDA-MB-231 cells. JAK, STAT3, PI3K, and mTOR relative expressions were measured using q-RT-PCR with GAPDH as a housekeeping gene. MDA-MB-231 cells treated with 5-FU for 72 h significantly increased the transcript levels of JAK, STAT3, PI3K, and mTOR compared to control cells. Data are presented as the mean ± SEM of three independent experiments. *** *p* < 0.001, * *p* < 0.05 compared with the control group.

Then, it was intriguing to unravel the resistance mechanism of 5-FU in MDA-MB-231 cells. The results showed that 5-FU markedly induced the transcript levels of JAK (*p* = 0.0141), STAT3 (*p* = 0.0167), PI3K (*p* = 0.0009), and mTOR (*p* = 0.0009) in MDA-MB-231 cells treated with 5-FU compared to control cells (Figure 2). This unravels a unique mechanism of resistance to 5-FU in MDA-MB-231 cells, as it was reported in other cancer cell lines that P38 MAPK [58], protein kinase CK2 [58], high-mobility group AT-hook 2 (HMGA2) [59], extracellular matrix 1 [60], and MDR proteins are among the altered proteins inducing 5-FU resistance in several malignant contexts. However, this is the first study representing such a novel resistance mechanism for 5-FU in MDA-MB-231 cell lines.

### 2.3. 5-FU Suppresses miR-1275, miR-615-5p, and Let-7i, Modulating PI3K/Akt/mTOR and JAK/STAT Pathways in MDA-MB-231 Cells

It was essential to unravel further the mechanism by which 5-FU induces the expression of JAK/STAT and PI3K/Akt/mTOR pathways in MDA-MB-231 cells to try to hijack such chemoresistance induced by 5-FU in TNBC cells. Six miRNAs (miR-155, miR-486-5p, miR-147, miR-1275, miR-615-5p, and Let-7i) that were validated to target JAK/STAT and PI3K/Akt/mTOR pathways either directly or indirectly were screened upon treatment of MDA-MB-231 cells by 5-FU.

**Figure 3:**
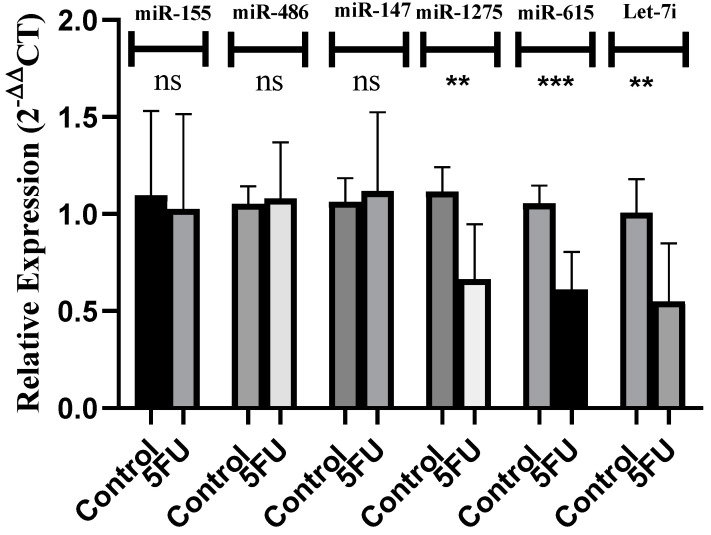
Impact of 5-FU on miRNAs regulating JAK/STAT and PI3K/Akt/mTOR pathways in MDA-MB-231 cells. Six miRNAs (miR-155, miR-486-5p, miR-147, miR-1275, miR-615-5p, and Let-7i) were measured relative to RNU6B (a housekeeping gene). MDA-MB-231 cells treated with 5-FU for 72 h resulted in a non-significant alteration of miR-155, miR-486-5p, and miR-147. However, it showed a significant attenuation of miR-1275, miR-615-5p, and Let-7i in 5-FU-treated cells compared to control cells. Data are presented as the mean ± SEM of three independent experiments. ^ns^ *p* > 0.05, ** *p* < 0.01, *** *p* < 0.001 compared with mock cells.

The results showed that miR-155, miR-486-5p, and miR-147 levels were not altered upon treatment of MDA-MB-231 cells by 5-FU (Figure 3). However, the expression levels of the tumor suppressor miRNAs miR-1275 (*p* = 0.0089), miR-615-5p (*p* = 0.0005), and Let-7i (*p* = 0.0050) were found to be markedly repressed in 5-FU-treated cells compared to control cells. This goes in line with an array of non-coding RNAs such as miR-320a [61], miR-135b, and miR-182 [62] that were evident to mediate chemoresistance in several malignant cell lines. However, this study represents a new evidence regarding the involvement of miR-1275, miR-615-5p, and Let-7i in inducing 5-FU chemoresistance in MDA-MB-231 cells, partially through their dominating effects in regulating PI3K/AKT/mTOR and JAK/STAT signaling pathways.

### 2.4. miR-1275, miR-615-5p, and Let-7i Are Tumor Suppressor miRNAs in TNBC

**Figure 4:**
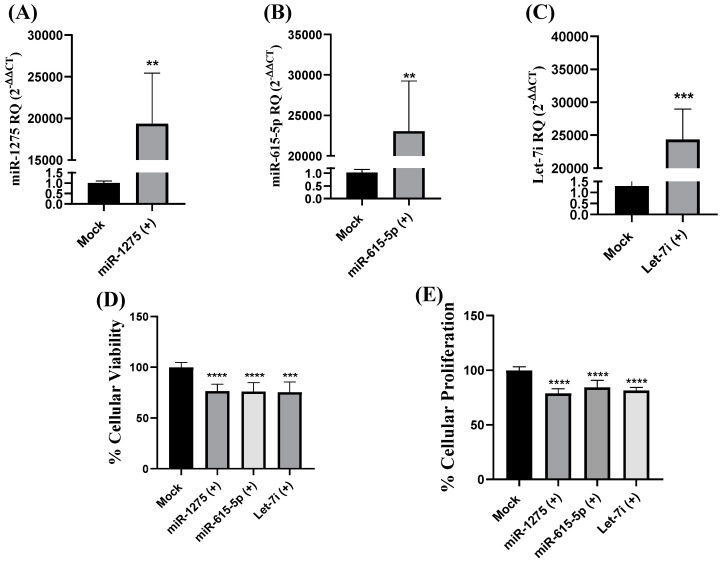
Impact of miR-1275, miR-615-5p, and Let-7i on cellular viability and proliferation in MDA-MB-232 cells. miR-1275, miR-615-5p, and Let-7i were measured relative to RNU6B (a housekeeping gene) in MDA-MB-231 cells transfected by respective oligonucleotide mimics and mock cells. Efficient and significant transfection of (**A**) miR-1275, (**B**) miR-615-5p, and (**C**) Let-7i was confirmed. (**D**) Cellular viability was assessed using the MTT assay. MDA-MB-231 cells transfected by miR-1275, miR-615-5p, and Let-7i showed significant repression in cellular viability compared to mock cells. Similarly, (**E**) the cellular proliferation rate was assessed using the BrdU incorporation assay. MDA-MB-231 cells transfected by miR-1275, miR-615-5p, and Let-7i showed significant repression in cellular proliferation compared to mock cells. Data are presented as the mean ± SEM of three independent experiments. ** *p* < 0.01, *** *p* < 0.001, **** *p* < 0.0001 compared with mock cells.

Next, it was essential to unravel the role of the respective miRNAs in TNBC cell lines. In such a context, MDA-MB-231 cells were transfected using miR-1275, miR-615-5p, and Let-7i mimics. Transfection efficiency was measured for each transfection, where miR-1275 showed more than a 19,000-fold increase in mimicked cells compared to mock cells. Similarly, miR-615-5p and Let-7i mimicked cells showed more than 23,000- and 24,000-fold increases in the levels of respective miRNAs compared to mock cells (Figure 4A–C). On the functional level, miR-1275, miR-615-5p, and Let-7i showed tumor suppressor effects through repressing cellular viability (*p* < 0.0001, *p* < 0.0001, and *p* = 0.0001, respectively) (Figure 4D) and cellular proliferation (*p* < 0.0001, *p* < 0.0001, and *p* < 0.0001, respectively) (Figure 4E). These results rank miR-1275, miR-615-5p, and Let-7i to act as tumor suppressor miRNAs in TNBC, such as miR-20a [63], miR-34a [45], miR-15/16 [64], miR-939-5p [14], and let-7a [35].

### 2.5. Average Sizes and Zeta Potential (ZP)

In a more translational approach, CsNPs were synthesized to act as a combo carrier for the three miRNA oligonucleotides inducing 5-FU resistance in TNBC cells in order to facilitate the translation of such a model into clinics. CsNPs would protect miRNA oligonucleotides from nucleases’ degradation in blood circulation, thus increasing their half-life time. Also, they are non-immunogenic and will not produce any immunotoxicity. Moreover, the stability of CsNPs under physiological conditions was confirmed using stability studies carried out in previously reported studies [32,55,65,66].

The ionic gelation technique was used to fabricate different CsNPs involving TPP crosslinkers, which lessens the mobility of the Cs structure and improves its physical and chemical features, including high stability and improved mechanical properties [67,68]. In addition, the ionic gelation was reported to enhance the thermo- and photoresistance of the prepared CsNPs as compared to free Cs. This is attributed to the formation of new inter- and intramolecular crosslinking interactions between the Cs amino groups and TPP, which minimize decomposition temperature [69].

**Figure 5:**
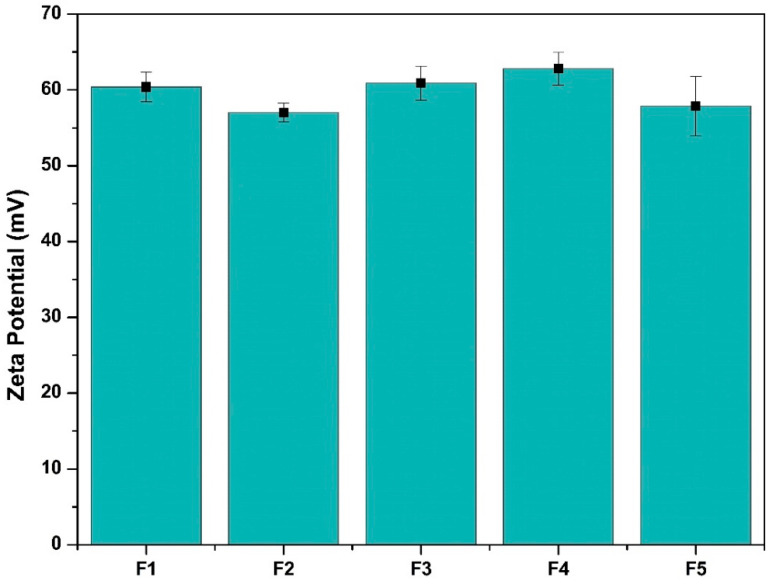
Zeta potential values of the prepared CsNP formulas.

In such a context, five different CsNPs were formulated where different cargos were used, as presented in Table 1. The dynamic light scattering technique was used to assess the average sizes of all formulated nanoparticles (Table 2) at 25 °C. All formulations had nanosizes lower than 270 nm. The average size after loading 5-FU (F1) was found to be 190.30 ± 6.3, which was increased to 209.1 ± 3.40, 206.30 ± 8.4, 216.40 ± 5.1 and 260.6 ± 11.5 after co-encapsulation of miR-1275 (F2), miR-615-5p (F3), Let-7i (F4) or the combo-3-miRNAs (F5), respectively. This demonstrated the effective integration of 5-FU and miRNA oligonucleotides into the CsNPs while maintaining their nanosizes. The formed sizes in the nanorange facilitate selective passive uptake into tumor cells via the leaky vasculature characterizing cancer cells [70]. The ZP of the loaded CsNP formulas is shown in Figure 5. All the zeta potential values were found in a narrow range between 57.00 ± 1.25 mV and 62.80 ± 2.17 mV. The high charge of the CsNP formulas is necessary to prevent the aggregation of the formed nanoparticles, rendering them more stable for a longer period of time [71].

Moreover, the highly positive charge would encourage the electrostatic interaction-boosted loading of the 5-FU and the miRNA oligonucleotides [52]. Furthermore, the high CsNPs’ charge would benefit the condensation/complexation of nucleic acids in these positively charged nanoparticles [53].

### 2.6. Morphology

**Figure 6:**
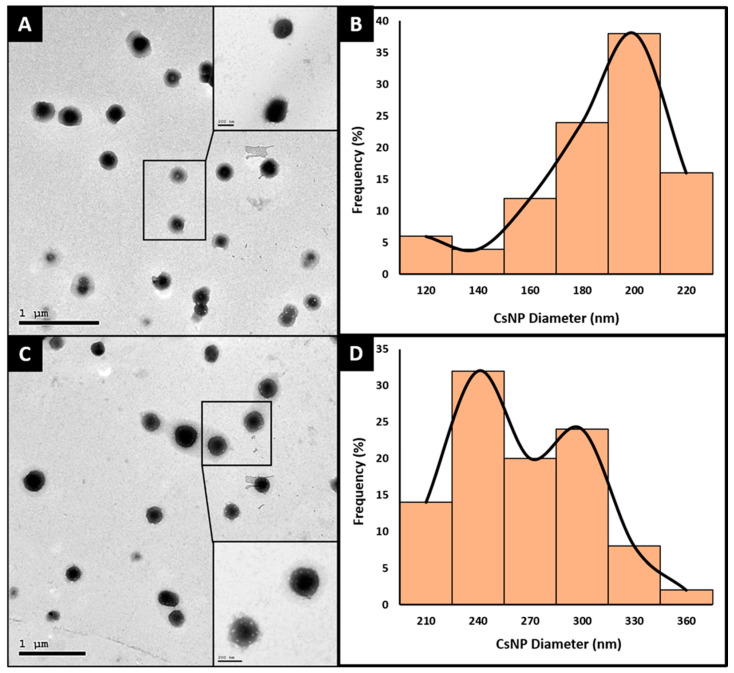
Transmission electron microscopy (TEM) images of (**A**) 5-FU-loaded CsNPs and (**C**) fluorouracil/miR-1275/miR-615-5p/Let-7i-loaded CsNPs with their diameter histograms (**B** and **D**, respectively).

To study the morphology and average sizes of CsNPs, TEM was carried out for the F1 and F5 formulae. F1 and F5 were the selected formulae for this characterization since they represent the two extremes of CsNP loading: F1 being the CsNPs loaded with 5-FU only, and F5 being the CsNPs loaded with 5-FU and all three miRNA oligonucleotides. The transmission electron micrographs of F1 and F5 are shown in Figure 6A and 6C, respectively. The CsNPs in both F1 and F5 formulae displayed a spherical morphology with minimal agglomeration. These findings align with the highly positive charge revealed by the zeta potential results. The CsNP charge elevation would enhance nanoparticles’ repulsion, hence reducing their aggregation. The average diameters of the selected F1 and F5 formulae were determined via ImageJ version 1.54g (NIH, Bethesda, MD, image processing software) and were found to be 176.67 ± 25.70 nm and 250.63 ± 37.00 nm, respectively, as presented in Figure 6A–D. These findings align very well with the data obtained from the DLS measurements. Also, F5 exhibited a significantly larger mean diameter (*p* < 0.001) compared to F1, owing to the condensation of the miRNA oligonucleotides inside the CsNPs. TEM images of the F1 and F5 formulas are presented in Figure 6A and 6C, respectively, and their diameter histograms are presented in Figure 6B and 6D, respectively.

### 2.7. Encapsulation Efficiency (EE%) and Drug Loading Capacity (DLC%)

The evaluation of EE% and DLC% of various therapeutic agents in NPs are essential parameters to assess nanoparticles’ biological efficiency. In such context, the EE% and DLC% of the five designed formulations (F1–F5) were subsequently studied, and the results are presented in Table 2. The highest EE% and DLC% of 5-FU (85.6 ± 2.1% and 2.9 ± 0.03, respectively) were observed in formula F5, implying that EE% and DLC% of 5-FU are correlated with the presence of the three highly negatively charged miRNAs together (miR-1275, miR-615-5p, and Let-7i) in formula F5. Relatively high miRNA oligonucleotides EE% and DLC% (more than 90% and 3.9%, respectively) were observed in the five formulations (F1-F5). This indicates that the three miRNAs’ oligonucleotides subjected to this investigation can be co-encapsulated with 5-FU into the CsNPs with high EE% and DLC% values. This is attributed to the high affinity of the negatively charged nucleotides in the miRNAs for the positively charged chitosan.

### 2.8. In Vitro Release Study

**Figure 7:**
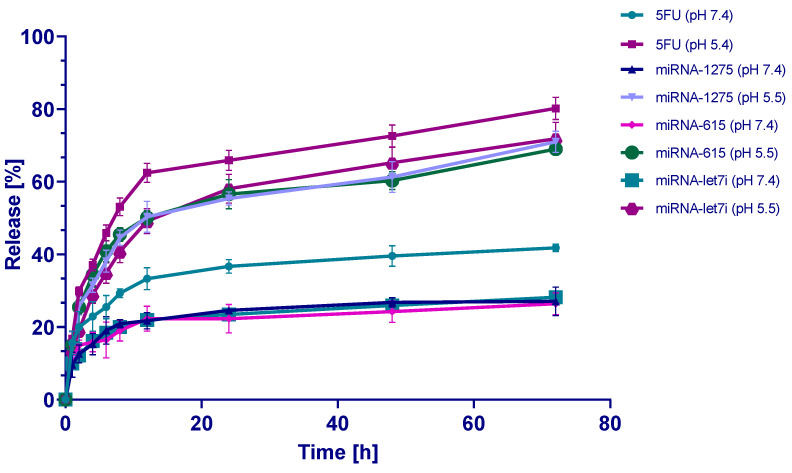
In vitro release assay. In vitro release of 5-FU, miR-1275, miR-615, and Let-7i from formula F5 in PBS at pH 7.4 and pH 5.5 at 37 °C, respectively.

The in vitro release manner of 5-FU, miR-1275, miR-615-5p, and Let-7i from F5 was assessed at 37 °C and at two different pH values (pH 7.4 and 5.5, respectively). As presented in Figure 7, approximately 33% and 60% of 5-FU were released from F5 within 24 h at pH 7.4 and 5.5, respectively, followed by sustained slow release where approximately 40% and 80% of 5-FU were released from F5 within 72 h at pH 7.4 and 5.5, respectively. On the other hand, the release of miR-1275, miR-615-5p, and Let-7i from F5 was much slower than 5-FU in both the physiological and acidic media. The faster release of 5-FU is attributed to its smaller molecular weight than the miRNA oligonucleotides, facilitating better release from the nanoparticles. On the other hand, the high negative charge of the nucleotides in the miRNAs possibly resulted in a strong electrostatic interaction with the cationic chitosan, resulting in a slower release of miR-1275, miR-615-5p, and Let-7i than 5-FU. Moreover, our findings showed that the release of 5-FU, miR-1275, miR-615-5p, and Let-7i at pH 5.5 was faster than that at pH 7.4, indicating that the acidic medium increases the release of the cargos from CsNPs compared to the physiological pH. These findings align very well with previous similar studies [72,73,74]. It is also worth noting that such acidic conditions simulate the malignant cells and their tumor microenvironment, while the neutral pH simulates the physiological cells [6]. Therefore, the preferential enhanced release of 5-FU and miR-1275, miR-615-5p, and Let-7i would be a promising therapeutic approach for improving the selectivity and efficiency of TNBC therapy.

The cationic amino groups in CsNPs are accountable for numerous of their developed functions, including selective release in tumor microenvironments and boosted biological activities [54]. The pH-dependent release of loaded therapeutic agents from CsNPs is essential in selective cancer therapy. At the physiological pH (7.4) of biological fluids, the glucosamine groups on the Cs surface are deprotonated. This leads to the deswelling of the CsNPs by lessening the intramolecular electrostatic repulsions inside the nanocomposite mesh [75].

On the other hand, the CsNPs became colloidally unstable at acidic pH (5.5) around the isoelectric point of the Cs–TPP NPs, which simulate the tumor microenvironment. This variation in ionic strength promoted critical structural changes that provoked swelling mechanisms initiated by osmotic pressures linked to the ionic distributions of the CsNPs. This leads to rapid disintegration due to the weakness of intramolecular interactions and, eventually, the pH-dependent release of cargo in the acidic tumor environment [75].

### 2.9. miR-1275, miR-615-5p, and Let-7i-Loaded CsNPs Abolished 5-FU Resistance in MDA-MB-231 Cells

**Figure 8:**
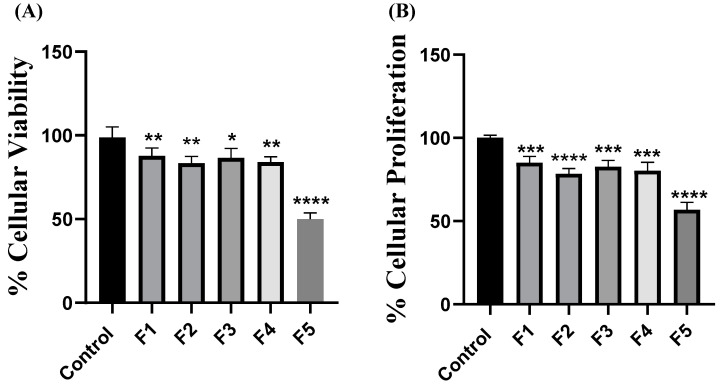
Impact of microRNA-loaded chitosan nanoparticles on MDA-MB-231 cellular viability and proliferation. (**A**) Cellular viability was assessed using MTT assay, where chitosan nanoparticles loaded with different cargos showed significant repression in cellular viability compared with control cells, where F5 showed the most significant repression in cellular viability. (**B**) The cellular proliferation rate was assessed using a BrdU incorporation assay. CsNPs loaded with different cargos showed significant repression in cellular proliferation compared with control cells, where F5 showed the most significant repression in cellular proliferation. Data are presented as the mean ± SEM of three independent experiments. * *p* < 0.05, ** *p* < 0.01, *** *p* < 0.001, **** *p* < 0.0001 compared with control cells.

As previously described in Table 1, formulated CsNPs were evaluated in MDA-MB-231 cells. The results showed that F1 (*p* = 0.0063), F2 (*p* = 0.0037), F3 (*p* = 0.0164), and F4 (*p* = 0.0014) repressed cellular viability (Figure 8A). However, F5 (*p* < 0.0001) showed synergistic activity and marked repression in cellular viability through alleviating the chemoresistance to 5-FU in MDA-MB-231 cells. Similarly, the results showed that F1 (*p* = 0.0007), F2 (*p* < 0.0001), F3 (*p* = 0.0001), and F4 (*p* = 0.0006) repressed cellular proliferation (Figure 8B). Yet, F5 (*p* < 0.0001) also showed the same synergistic activity and marked repression in cellular proliferation through alleviating the chemoresistance to 5-FU in MDA-MB-231 cells. This could be attributed to the synergistic activity of miRNA oligonucleotides in F5 in markedly abrogating 5-FU resistance in MDA-MB-231 cells, partially through counteracting the activation of the Pi3K/Akt/mTOR and JAK/STAT pathways. However, in the case of F1–F4, a significant reduction in cellular viability and proliferation was observed due to the respective miRNA effects of either miR-1275, miR-615-5p, or Let-7i alone. Nevertheless, it was not as potent as F5 in repressing cellular viability and proliferation of MDA-MB-231 cells due to the lack of cumulative effects of miR-1275, miR-615-5p, and Let-7i oligonucleotides. It is also worth noting that since miRNAs, specifically miR-1275, miR-615-5p, and Let-7i, have been thoroughly reported to modulate other oncogenic signaling cascades such as insulin-like growth factors and Wnt/β-catenin signaling pathways, this could be another possible reason for the additive effects witnessed in F5 and not in F2–F4.

## 3. Materials and Methods

### 3.1. Materials, Cell Culture, and Treatment

Low-molecular-weight chitosan was purchased from Biosynth, Carbosynth, Berkshire, UK. TPP (sodium tripolyphosphate), glacial acetic acid, and 5-Fluorouracil (5-FU) were purchased from Sigma-Aldrich, St. Louis, MO, USA.

MDA-MB-231 adherent TNBC cell lines (purchased from ATCC, Manassas, VA, USA, HTB-26™) were utilized in the study. MDA-MB-231 cells showed the highest activation profiles of PI3K/Akt/mTOR and JAK/STAT oncogenic pathways compared to other MCF-7 and Bt-474 BC cells. Nonetheless, it has the highest metastatic potential and aggressive behavior. They were cultured in Dulbecco’s modified Eagle’s medium (DMEM; Lonza, Basel, Switzerland) enriched and supplemented with 4.5 g/L glucose, 4 mmol/L L-glutamine, 10% fetal bovine serum (FBS) (Biowest, Nuaillé, France), MycoZap (1:500; Lonza, Köln, Germany), and 1% penicillin/streptomycin (Lonza) at 37 °C in a 5% CO_2_ atmosphere. When the cells were 70–80% confluent, they were passaged [65,76,77]. Cells were either treated by 5-FU (3.84 µmol/L) for 72 h, formulated CsNPs, or transfected for 48 h by respective oligonucleotides.

### 3.2. Cellular Viability Assay (MTT)

The 3-(4, 5-dimethylthiazol-2-yl)-2,5diphenyltetrazolium bromide (MTT) assay was utilized for the cellular viability studies. Ten thousand MDA-MB-231 cells were seeded in 200 µL of media in a 96-well plate and incubated for 24 h before transfection of the miRNA mimics. The media were removed 48 h post-transfection or 72 h post-treatment, and 20 µL of working solution (MTT solution in phosphate-buffered saline) (Sigma Aldrich, Darmstadt, Germany) was added to each well. After 5–6 h, formazan was re-suspended and solubilized using 200 µL dimethylsulfoxide (DMSO) (Riedel de Haen, Hanover, Germany), and then absorbance was measured at 480 nm [35].

### 3.3. Cellular Proliferation Assay (BrdU Assay)

The bromodeoxyuridine (BrdU) incorporation assay was utilized for the proliferation studies. Black 96-well plates were used to seed TNBC cells, with an initial cell count of 5 × 10^4^ cells/well, and incubated for 24 h before transfection or treatment. Following transfection/treatment, the Cell Proliferation ELISA Kit (Roche Applied Science, Penzberg, Germany) was used to label the cells for 4 h with BrdU labeling reagent. After that, the cells were fixed for 30 min with Fix-Denate and further incubated for 90 min with Anti-BrdU POD, and then measurements were performed [78].

### 3.4. Transfection of BC Cell Lines with Different Oligonucleotides

MDA-MB-231 TNBC cells were transfected using different miRNA oligonucleotides such as miR-1275, Let-7i, and miR-615-5p mimics and their scrambled miRNAs as controls using HiPerfect Transfection Reagent (Qiagen, Hilden, Germany) as described in previous experiments [35].

### 3.5. Total RNA Extraction from TNBC Cell Lines

Biazol reagent (Invitrogen, Carlsbad, CA, USA) was used to extract total RNA from MDA-MB-231 TNBC cells. A spectrophotometer was used to measure the amount of RNA produced, and 1% agarose gel electrophoresis was used to detect 18s rRNA bands to ensure RNA purity and integrity, respectively. RNA samples with an optical density ratio 260/280 higher than 2 were excluded from the study [63]. In the case of transfection using miRNA oligonucleotides, 48 h post-transfection, RNA is extracted from MDA-MB-231 cells transfected by either miRNA oligonucleotides labeled as “miR-1275 (+)/miR-615-5p (+)/Let-7i (+)” or transfected by transfection reagent only labeled as “Mock”. In the case of CsNPs or 5-FU treatment, 72 h post-treatment, RNA is extracted from MDA-MB-231 cells treated by either any of the respective CsNPs formulas, thus labeled as (F1–F5) or transfected by a vehicle only labeled as “Control”.

### 3.6. Quantitative Real-Time Polymerase Chain Reaction (qRT-PCR) Analysis

The extracted miRNAs were reverse-transcribed into the corresponding single-stranded cDNA using the TaqMan miRNA reverse transcription kit (ABI, Los Angeles, CA, USA) along with specific primers Let-7i, miR-615-5p, miR-1275, miR-155, miR-147, miR-486-5p, and RNU6B (as a housekeeping gene). TaqMan Real-Time qPCR on StepOne^TM^ Systems was used to quantify all cDNAs (ABI). Using the 2^−ΔΔCT^ method, the relative quantification values were calculated [16,79].

### 3.7. Preparation of CsNPs

Drug-loaded CsNPs were prepared via the coacervation (or ionic gelation) method. Sodium tripolyphosphate (TPP) was used as the crosslinker in the coacervation technique. Chitosan was dissolved (1 mg/mL) in 1% (*v*/*v*) glacial acetic acid, then filtered with syringe filters of 0.45 µm pore-sized filters. The solution was left on a magnetic stirrer. After stirring for 24 h, 5-FU was added to reach a concentration of 0.5 mg/mL, and the different miRNA oligonucleotides were also added to reach a concentration of 1 µg/mL. The loading components of the different CsNP formulas are presented in Table 1. Consequently, 200 µL of the TPP crosslinker (1 mg/mL) was mixed with the loaded cargos and then added dropwise to 5 mL of the chitosan solution under rapid magnetic stirring. Then, the nanoparticle colloid was left on the stirrer for 30 min [80].

### 3.8. Characterization of CsNPs

The average sizes and zeta potential (ZP) of the designed CsNPs were measured using the laser Doppler velocimetry (LDV) technique via the Malvern ZEN 3600 Zetasizer (Malvern Instruments, Worcestershire, UK). Before measurements, all the samples were sonicated for 5 min. All measurements were performed at room temperature in triplicates. The results are presented as the mean value ± standard deviation. The average sizes and morphologies of the prepared CsNPs were determined using transmission electron microscopy (TEM, JEOL-JEM 2100 electron microscope, Musashino, Akishima, Tokyo, Japan) operating at 160 kV. The image processing program ImageJ version 1.54g (NIH, Bethesda, MD, USA) was used to construct a histogram demonstrating the CsNPs’ size from 50 measurements.

### 3.9. Encapsulation Efficiency (%)

To study the encapsulation efficiency of 5-FU, miR-1275, miR-615-5p, and Let-7i in the different prepared formulations (F1, F2, F3, F4, and F5), the formulations were centrifuged at 12,000 rpm for 2 h at 4 °C. Then, the unloaded 5-FU and/or miRNA oligonucleotides were retrieved from the supernatant. The amounts of unloaded 5-FU, miR-1275, miR-615-5p, and Let-7i in the supernatant were quantified employing UV–Vis spectrophotometry (Peak instruments T-9200, Houston, TX, USA) at 289, 310, 267, and 255 nm, respectively. The EE (%) was calculated employing the equation below [71,81,82].

$$EE\;\%=\frac{Initial\;amount\;of\;drug-the\;amount\;of\;unloaded\;drug\;}{Initial\;amount\;of\;drug}\times 100$$

In addition, the drug loading capacity percent (DLC%) was computed using the equation below:

$$\;DLC\%=\frac{Amount\;of\;loaded\;drug}{Initial\;amount\;of\;drug+Initial\;amount\;of\;polymer}\times 100$$

### 3.10. Release Study

To study the release manner of 5-FU, miR-1275, Let-7i, and miR-615-5p from formula F5, the NPs were suspended in 2 mL of deionized water and loaded into dialysis tubes (50 KD cut-off). Then, the dialysis tube was dipped in 15 mL of dialysis release solution (phosphate-buffered saline, PBS of pH 7.4 and 5.5). The release medium was fortified with sodium lauryl sulfate (5%) to improve the solubility of hydrophobic drugs and minimize release resistance. Then, the system was placed in a vibrating incubator (Jeio tech SI-300, Seoul, Republic of Korea) at 37 ± 1 °C and a shaking speed of 140 rpm. At indicated time intervals over 72 h, the released 5-FU, or miRNA oligonucleotides, were monitored in the release medium. The concentrations of the released 5-FU, miR-1275, Let-7i, and miR-615-5p were estimated by UV–Vis spectrophotometry (as described previously) and immediately replaced with an equal volume of fresh medium to maintain sink conditions. The release percentage was estimated using the equation below [83,84].

$$Release\%=\frac{Amount\;of\;released\;drug}{Initial\;amount\;of\;loaded\;drug}\times 100$$

## 4. Conclusions

**Figure 9:**
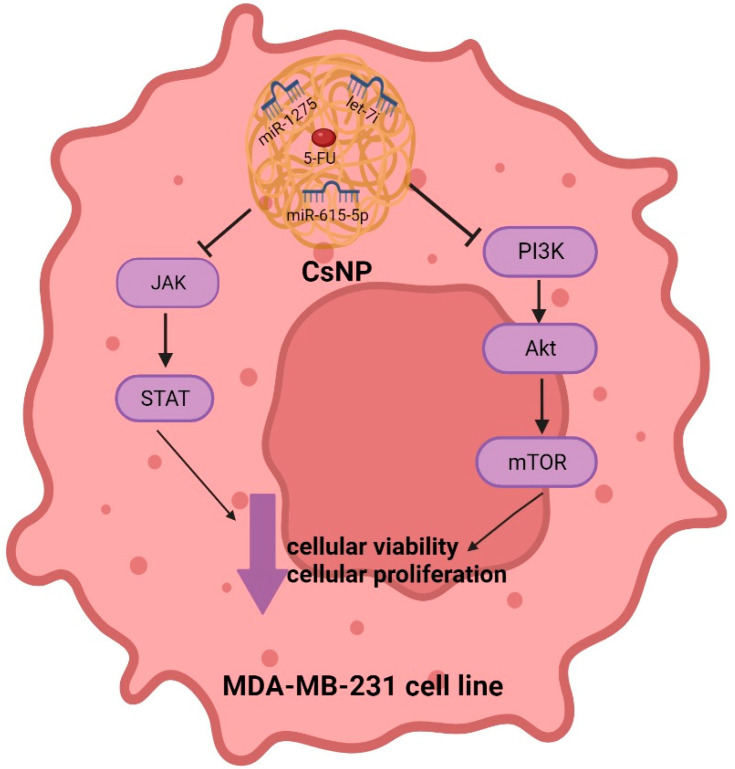
Graphical representation of F5-CsNPs effects in MDA-MB-231 cells.

This study highlights a novel molecular resistance mechanism for 5-FU in MDA-MB-231 cells. PI3K/Akt/mTOR and JAK/STAT oncogenic signaling cascades were proven to be induced upon 5-FU treatment of MDA-MB-231 cells. This study revealed that the induction of oncogenic signaling pathways was mediated through co-treatment with miRNAs. The tumor suppressor miRNAs (miR-1275, miR-615-5p, and Let-7i) were outlined for the first time in this study as fundamental cornerstones of 5-FU chemoresistance in TNBC cells. Moreover, this study provides a novel therapeutic approach to hijacking 5-FU chemoresistance in TNBC patients using a novel CsNP system co-loaded with miRNA oligonucleotides that successfully resulted in a marked abrogation of 5-FU resistance in MDA-MB-231 cells (Figure 9).

This figure represents a summary of the efficiency of CsNPs loaded with 5-FU and miRNAs: miR-1275, miR-615-5p, and Let-7i to abolish 5-FU resistance in MDA-MB-231 cells partially through repressing PI3K/Akt/mTOR and JAK/STAT pathways. The abolishment of 5-FU resistance was confirmed by significant repression of MDA-MB-231 cellular viability and proliferation.

## Figures and Tables

**Table 1:** Loading components of the different CsNP formulas.

Formula Code	5-FU	miR-1275	miR-615-5p	Let-7i
F1	+	−	−	−
F2	+	+	−	−
F3	+	−	+	−
F4	+	−	−	+
F5	+	+	+	+

**Table 2:** Average sizes and EE% and DLC% of the five prepared formulas. Data are presented as the mean ± SD; n = 3.

Formula	Average Size (nm)	EE [%]				DLC [%]			
		5-FU	miR-1275	miR-615-5p	Let-7i	5-FU	miR-1275	miR-615-5p	Let-7i
F1	190.3 ± 6.3	70.3 ± 2.1	-	-	-	2.3 ± 0.1	-	-	-
F2	209.1 ± 3.4	79.8 ± 3.9	91.1 ± 2.4	-	-	2.6 ± 0.2	3.3 ± 0.1	-	-
F3	206.3 ± 8.4	80.7 ± 4.2	-	93.6 ± 2.7	-	2.5 ± 0.05	-	3.5 ± 0.17	-
F4	216.4 ± 5.1	78.5 ± 2.6	-	-	92 ± 4.9	2.7 ± 0.1	-	-	3.4 ± 0.02
F5	260.6 ± 11.5	85.6 ± 2.1	96.3 ± 1.9	98.5 ± 3.3	97.1 ± 4.1	2.9 ± 0.03	3.9 ± 0.1	4.1 ± 0.08	4.0 ± 0.07

## Data Availability

Data are contained within this article.
